# Liver Metabolomics and Inflammatory Profiles in Mouse Model of Fentanyl Overdose Treated with Beta-Lactams

**DOI:** 10.3390/metabo13080965

**Published:** 2023-08-21

**Authors:** Fawaz Alasmari, Mohammed S. Alasmari, Mohammed A. Assiri, Mohammed Alswayyed, Syed Rizwan Ahamad, Abdulrahman I. Alhumaydhi, Bandar I. Arif, Sahar R. Aljumayi, Abdullah F. AlAsmari, Nemat Ali, Wayne E. Childers, Magid Abou-Gharbia, Youssef Sari

**Affiliations:** 1 Department of Pharmacology and Toxicology, College of Pharmacy, King Saud University, Riyadh 11451, Saudi Arabia; 2 Department of Pathology and Laboratory Medicine, College of Medicine, King Saud University, Riyadh 11451, Saudi Arabia; 3 Department of Pharmaceutical Chemistry, College of Pharmacy, King Saud University, Riyadh 11451, Saudi Arabia; 4 Department of Pharmaceutical Sciences, Temple University School of Pharmacy, Philadelphia, PA 19140, USA; 5 Department of Pharmacology and Experimental Therapeutics, College of Pharmacy and Pharmaceutical Sciences, University of Toledo, Toledo, OH 43606, USA

**Keywords:** opioids, overdose, beta-lactams, ceftriaxone, MC-100093, metabolomic, inflammation, IL-6, CYP-3A4

## Abstract

Fentanyl is a highly potent opioid analgesic that is approved medically to treat acute and chronic pain. There is a high potential for overdose-induced organ toxicities, including liver toxicity, and this might be due to the increase of recreational use of opioids. Several preclinical studies have demonstrated the efficacy of beta-lactams in modulating the expression of glutamate transporter-1 (GLT-1) in different body organs, including the liver. The upregulation of GLT-1 by beta-lactams is associated with the attenuation of hyperglutamatergic state, which is a characteristic feature of opioid use disorders. A novel experimental beta-lactam compound with no antimicrobial properties, MC-100093, has been developed to attenuate dysregulation of glutamate transport, in part by normalizing GLT-1 expression. A previous study showed that MC-100093 modulated hepatic GLT-1 expression with subsequent attenuation of alcohol-increased fat droplet content in the liver. In this study, we investigated the effects of fentanyl overdose on liver metabolites, and determined the effects of MC-100093 and ceftriaxone in the liver of a fentanyl overdose mouse model. Liver samples from control, fentanyl overdose, and fentanyl overdose ceftriaxone- or MC-100093-treated mice were analyzed for metabolomics using gas chromatography–mass spectrometry. Heatmap analysis revealed that both MC-100093 and ceftriaxone attenuated the effects of fentanyl overdose on several metabolites, and MC-100093 showed superior effects. Statistical analysis showed that MC-100093 reversed the effects of fentanyl overdose in some metabolites. Moreover, enrichment analysis revealed that the altered metabolites were strongly linked to the glucose-alanine cycle, the Warburg effect, gluconeogenesis, glutamate metabolism, lactose degradation, and ketone body metabolism. The changes in liver metabolites induced by fentanyl overdose were associated with liver inflammation, an effect attenuated with ceftriaxone pre-treatments. Ceftriaxone normalized fentanyl-overdose-induced changes in liver interleukin-6 and cytochrome CYP3A11 (mouse homolog of human CYP3A4) expression. Our data indicate that fentanyl overdose impaired liver metabolites, and MC-100093 restored certain metabolites.

## 1. Introduction

Opioids exert their pharmacological effects by interacting with opioid receptors in the brain and peripheral tissues [1,2,3,4]. There are opioids that stimulate opioid receptors, such as those that are naturally isolated from poppy seeds, semi-synthetics, and synthetics [5]. Opioids are commonly used to alleviate moderate-to-severe pain because of their analgesic and sedative effects [6,7,8,9]. Opioid misuse can cause respiratory depression and consequently death through the overstimulation of peripheral and central opioid receptors [10,11]. Recent studies have shown that opioid overdoses cause a significant number of deaths globally [11,12]. Naloxone, an opioid receptor antagonist, is available in emergency rooms and can rapidly reverse the effects of opioid overdoses [13]. However, naloxone should be initiated at an appropriate dosage for the therapeutic ratio and time of monitoring patients [14]. Therefore, investigating the dysregulated molecular pathways involved in opioid overdoses is essential to determine potential therapeutic strategies for the treatment of opioid overdoses and to prevent the development of liver toxicity and other organ injuries.

Pharmaceutical companies developed fentanyl as an effective and rapid-acting analgesic with great potency. Fentanyl has the greatest therapeutic ratio and quickest time of action as compared to other opioids [15]. Moreover, fentanyl is more lipophilic and potent, being approximately 50–100 times more lipophilic than morphine [15]. Fentanyl is available in many dosage forms, including parenteral formulations and transdermal patches [16]. The recent rise in fentanyl overdoses is mainly driven by illicitly manufactured fentanyl (IMF), rather than pharmaceutical fentanyl. IMF comes in a variety of dosage forms in the drug market, including liquids and powders. Owing to its tremendous potency, it is frequently mixed with other drugs, making them more effective and addictive [16].

In this study, we used metabolomic analysis as a methodological tool to determine biomarkers in tissues and bodily fluids that are mainly end- or intermediate metabolites of different biological pathways, including amino acids, carbohydrates, and sugars [17]. Study demonstrated that heroin self-administration was associated with significant alterations in different metabolomic biomarkers, such as phospholipids and intermediates in the Tricarboxylic Acid Cycle in rat serum [18]. A clinical study from our laboratory found that patients with amphetamine- or cannabis-use disorder had dysregulated serum metabolites compared to the healthy control group [19]. Another clinical study found that individuals with opioid-use disorder (OUD) had 712 metabolomic peaks that differentiated them from OUD-negative individuals using ultra-performance liquid chromatography and high-resolution mass spectrometry [20]. In the current study, we used gas chromatography–mass spectrometry (GC-MS) to explore the effects of fentanyl overdose on liver metabolites in mice. Our study focused on selected metabolites that previously were investigated in patients diagnosed with amphetamine- or cannabis-use disorder [19]. In addition, a previous study reported that during reinstatement to heroin-seeking behavior, there were changes in the concentrations of amino acids, fatty acids, and precursors of neurotransmitters, ketone bodies, and glucose in the serum of rats [20]. Furthermore, another study investigated the effects of opioids (morphine and heroin) on metabolomic profiles, including amino acids, lipids, and carbohydrates, in the serum and urine of mice during repeated exposure, withdrawal, and relapse conditions [21]. However, little is known about the modulatory effects of opioids overdose on amino acid, fatty acids, and sugars, as well as liver injury. In this study, we investigated the effects of fentanyl overdose on selected metabolites and histopathological changes and inflammatory markers in mouse liver.

Furthermore, we tested the effects of beta-lactams against fentanyl-overdose-induced liver injury. Beta-lactams are potent upregulators of glutamate transporter (GLT-1) expression, by increasing transcription of GLT-1 gene in the mesocorticolimbic brain regions [22,23,24]. Beta-lactams have been extensively studied for their neuroprotective and drug-dependence-attenuating effects by modulating glutamate homeostasis in animal models [25,26,27,28]. For example, ceftriaxone was effective in attenuating Alzheimer’s disease, stroke, and substances of abuse-seeking behaviors, at least in part, by increasing GLT-1 expression in several brain regions in rodents [27,28,29,30]. Upregulation of GLT-1 attenuated ethanol-increased extracellular glutamate concentration in the nucleus accumbens [31]. MC-100093, a synthetic beta-lactam compound, was found to attenuate cocaine- and alcohol-seeking behaviors by upregulating GLT-1 in the brain [32,33]. Importantly, MC-100093 normalized GLT-1 expression and PPAR-a and attenuated ethanol-increased fat lipid content in the livers of rats exposed to ethanol for five weeks [32]. In the present study, we investigated the modulatory effects of MC-100093 and ceftriaxone on liver metabolites in mice exposed to fentanyl overdose.

**Figure 1:**
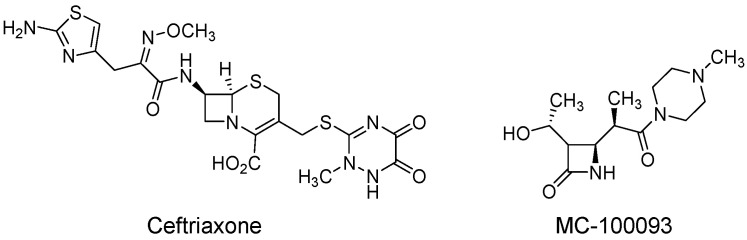
Structures of ceftriaxone and MC-100093.

One of the advantages of MC-100093 is the lack of antibacterial effects, which avoids the occurrence of antibiotic resistance or a number of antibiotic-related side effects (e.g., diarrhea) associated with the chronic use or misuse of beta-lactam antibiotics. In addition, MC-100093 is superior not only due to the advantages mentioned above but also in terms of pharmacokinetic parameters (ADME) as compared to ceftriaxone. Although MC-100093 is more hydrophilic, it has reasonable oral bioavailability in rats (28%), whereas ceftriaxone is not orally bioavailable [33]. Moreover, MC-100093 crosses the blood–brain barrier to a greater degree (brain/plasma ratio of 14% in mice) than ceftriaxone (brain/plasma ratio of 1% in mice) [34]. Another advantage is that it does not significantly inhibit the major cytochrome P450 enzymes (e.g., CYP3A4, CYP2C9, CYP2D6) responsible for the metabolism of the majority of drugs used in clinical practice [33]. MC-100093 was found to be up to 10 times more potent than ceftriaxone in increasing glutamate uptake in astrocytes [33]. The chemical structures of ceftriaxone and MC-100093 are shown in Figure 1.

Our present study, for the first time, investigated the effects of beta-lactam compounds on the metabolomics pathways in the livers of mice exposed to repeated low doses and a final high dose of fentanyl. Our study also identified possible signaling pathways involved in fentanyl-overdose-dysregulated metabolites in the liver. These metabolites might be affected by opioid overdose through the alteration of glutamate metabolism. In addition, other signaling pathways, such as gluconeogenesis and lactose degradation, may also be modulated by opioid overdose.

## 2. Materials and Methods

### 2.1. Animal Use Approval

This study was approved by the Institutional Animal Care and Use Committee (IACUC) of the Research Ethics Committee of King Saud University (ethics reference number KSU-SE-22-48).

### 2.2. Study Design

Male BALB/c mice were divided into four groups at age of 7–8 weeks: (1) control group (*n* = 4); (2) fentanyl group (*n* = 5); (3) fentanyl–ceftriaxone group (*n* = 5); and (4) fentanyl-MC-100093 group (*n* = 6). Groups 2, 3, and 4 were injected with fentanyl (50 µg/kg, i.p.) on days 1, 3, 5, and 7. The dosing protocol of intermittent exposure to fentanyl was applied in our study to mimic the human opioid exposure as described previously [35]. On day 9, these mice were i.p. injected with fentanyl at a dose of 1 mg/kg. The control group was i.p. injected with normal saline on the corresponding days. In addition, groups 3 and 4 were injected daily with ceftriaxone (200 mg/kg, i.p.) and MC-100093 (50 mg/kg, i.p.), respectively, from days 5 to 9. Groups 1 and 2 were i.p. injected with normal saline on days 5–9. On day 10, mice were sacrificed, and liver samples were collected and stored at −80 °C for metabolomic analysis.

### 2.3. Gas Chromatography–Mass Spectrometry (GC-MS)

#### 2.3.1. Chemicals

Pyridine, bis-N,O-trimethylsilyl trifluoroacetamide (BSTFA), and methoxyamine hydrochloride were obtained from Sigma-Aldrich (St. Louis, MO, USA). Methanol and hexane were obtained from BDH VWR International Ltd. (Poole, UK). Deionized water was obtained using a Milli-Q Plus device (Millipore, Billerica, MA, USA).

#### 2.3.2. Sample Preparation

Frozen liver samples were defrosted and weighed at room temperature. After 5 min, the tissue was transferred into a 10 mL Corning centrifuge tube and 5 mL of methanol was added. A tissue homogenizer was used to dissolve the samples in methanol. The tubes were centrifuged for 5 min at 10,000 rpm at 4 °C. The supernatant (200 µL) from the centrifuge was transferred into a 2 mL GC-MS vial. The solution was then dried using nitrogen purging. Next, 100 µL of methoxyamine hydrochloride (20 mg/mL) was added to the pyridine solution. This mixture was vortexed for 10 min and incubated at room temperature for 16 h. The derivatization procedure was then carried out on the methoxymate sample using 100 µL BSTFA/TMCS (99/1, *v*/*v*), vortexed once more for 10 min, and maintained at 50 °C for 2 h. The derivatized sample (1 µL) was injected using a split-mode injection device (split ratio of 1:20) [36,37].

#### 2.3.3. Parameter Description

Liver samples were analyzed using gas chromatography–mass spectrometry (GC-MS) using a PerkinElmer Clarus 600 gas chromatograph, Clarus 600 T mass spectrometer, and Turbomass software, as described previously (8, 9). An aliquot (1 µL) of the derivatized sample was taken using a GC/MS system with an elite 5MS column (length: 30.0 m, inner diameter of 0.25 mm, and thickness of 0.5 µm (PerkinElmer, Waltham, MA, USA). The mobile phase in this experiment was He gas supplied at a flow rate of 1.0 mL/min. Fused silica was used as the stationary phase. The injector temperature was 280 °C. The initial oven temperature of the GC-MS system was held at 40 °C for 2 min, which was increased to 150 °C at a heating rate of 10 °C, held for 2 min, and then increased to 300 °C at the same heating rate, and finally held for 2 min. The overall run time was 32 min. The injector and interface temperatures were set to 220 °C and 240 °C, respectively. Electron ionization was employed for MS detection at scanning rates of 50–600 *m*/*z*. The multiplier voltage was maintained at 330 V, whereas the electron energy was maintained at 70 eV. The solvent delay was 7 min owing to pyridine and the derivatizing agent. Unknown compounds were identified through spectral comparisons using the National Institute of Standards and Technology (NIST) library (2005) and Wiley library (2006).

### 2.4. Histopathology

Livers were dissected and immediately stored in 4% formaldehyde, and paraffin-embedded later. A microtome was used for liver sectioning; the sections were then stained with hematoxylin and eosin (H&E). To assess the extent of liver injury, the morphology of hepatocyte, including liver parenchyma, central vein, and portal tract were compared in all groups using an optical microscope (Olympus BX microscope and DP72 camera, Melville, FL, USA). Inflammatory cells were analyzed using the microscope.

### 2.5. Western Blot Assay

Western blot assay was performed according to our previous study [38]. Liver tissues from the four groups were homogenized in RIPA lysis buffer (phosphatase and protease inhibitor). Protein assay (BCA assay kit) was conducted, and equal amounts of protein were then separated using sodium dodecyl sulphate–polyacrylamide gels and electrophoresis (SDS–PAGE). Using transfer equipment, separated proteins were transferred into polyvinylidene difluoride (PVDF) membranes. After blocking membranes with 3% non-fat milk in 1 tris buffered saline with tween (TBST) for 1 h, primary antibodies were then incubated overnight at 4 °C. These primary antibodies were rabbit anti-interleukin-6 (IL-6, MyBiosource, San Diego, CA, USA), rabbit anti-CYP3A11 (mouse homolog of human CYP3A4) (ABclonal, Wuhan, China), and rabbit anti-β-actin (ABclonal, Wuhan, China). The PVDF membranes were incubated with the secondary antibody in 3% non-fat milk in 1 TBST for 90 min the next day. In order to identify the band signals, a chemiluminescence-detecting reagent was applied, and imaging equipment (ChemiDocTMMP-Bio-Rad, Bio-Rad, Hercules, CA, USA) was used. The quantification of protein expression was then performed using Image J program (Java 8).

### 2.6. Statistical Analysis

The metabolomic profiles of the control, fentanyl, fentanyl–ceftriaxone, and fentanyl-MC-100093 groups were analyzed statistically using GraphPad Prism software. To determine the significance of the metabolite peak area ratios among the different groups, a one-way ANOVA test was conducted. For each metabolite, the ratio of the peak area was determined by dividing the metabolite peak area by the average peak area of the metabolite of the control group. All the values were used without excluding outliers. To determine any statistical differences in metabolites between the four groups, Holm–Sidak’s multiple comparisons test was employed. One-way ANOVA followed by Holm–Sidak’s multiple comparisons was also used to investigate the changes in IL-6 and CYP3A11 liver expression using Western blot assay between all groups (the groups were normalized to the 100% of the control group). *p* ≤ 0.05 was set as a level of significance.

### 2.7. Heatmap and Enrichment Analysis Model

The MetaboAnalyst program was used to identify potential signaling pathways involved in metabolomic changes in the fentanyl-overdosed mouse model. The software was used to further analyze the metabolomic profile using partial least-squares-discriminant analysis (PLS-DA), heatmap analysis, scores plot, and metabolite set enrichment analysis. The metabolites used to generate the enrichment analysis were significantly different between the control and fentanyl groups, or between any treatment and fentanyl groups, but were not significant between the treatment and control groups. However, only beta-prostaglandins and allonic acids were not investigated for enrichment analysis.

## 3. Results

### 3.1. Overall Metabolomic Profiling, Heatmap, Partial Least-Squares-Discriminant Analysis (PLSDA), Peak Ratio of Selected Metabolites of Fentanyl, Fentanyl–Ceftriaxone, and Fentanyl-MC-100093 Groups

#### 3.1.1. Overall Metabolomic Profiling of Fentanyl, Fentanyl–Ceftriaxone, and Fentanyl-MC-100093 Groups

**Figure 2:**
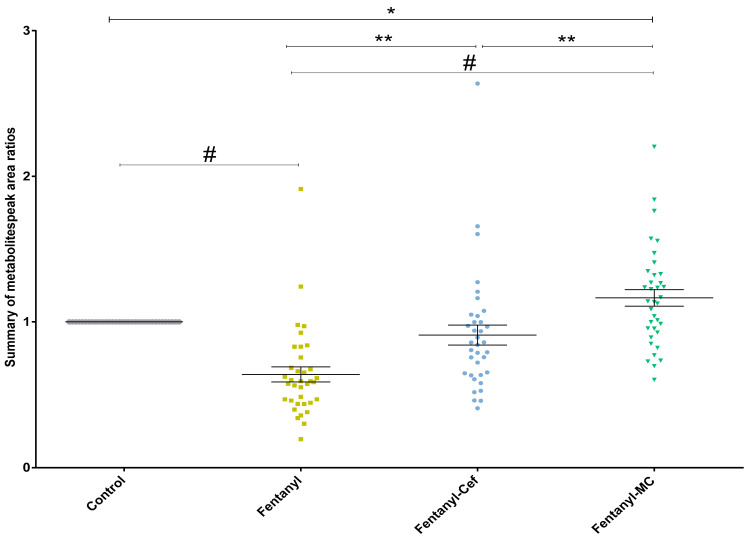
Overall metabolomics profiles in fentanyl, fentanyl-MC-100093, and fentanyl–ceftriaxone groups. One-way ANOVA showed significant changes in the metabolomic profiles in fentanyl, fentanyl-MC-100093, and fentanyl–ceftriaxone groups. One-way ANOVA followed by Holm–Sidak’s multiple comparisons test showed significant differences in metabolomics profiles between the fentanyl group and the other three groups. The analysis also found significant differences in metabolomics profiles between fentanyl-MC-100093 and fentanyl–ceftriaxone groups. Each dot in the graph represents the mean of one metabolite in each group. Data are reported as the mean of all metabolites’ means ± SEM. (* *p* < 0.05, ** *p* < 0.01, # *p* < 0.0001, *n* = 4–6/group). Cef, ceftriaxone; MC, MC-100093.

One-way ANOVA showed significant changes in metabolomic profiles in the fentanyl, fentanyl-MC-100093, and fentanyl–ceftriaxone groups. One-way ANOVA followed by Holm–Sidak’s multiple comparisons test showed significant differences in the overall metabolic profiles between the fentanyl group and the other three groups (Figure 2). The analysis also revealed significant differences in the metabolomic profiles between the fentanyl-MC-100093 and fentanyl–ceftriaxone groups, and between the control and fentanyl-MC-100093 groups (Figure 2).

#### 3.1.2. Heatmap of Fentanyl, Fentanyl–Ceftriaxone and Fentanyl-MC-100093 Groups

**Figure 3:**
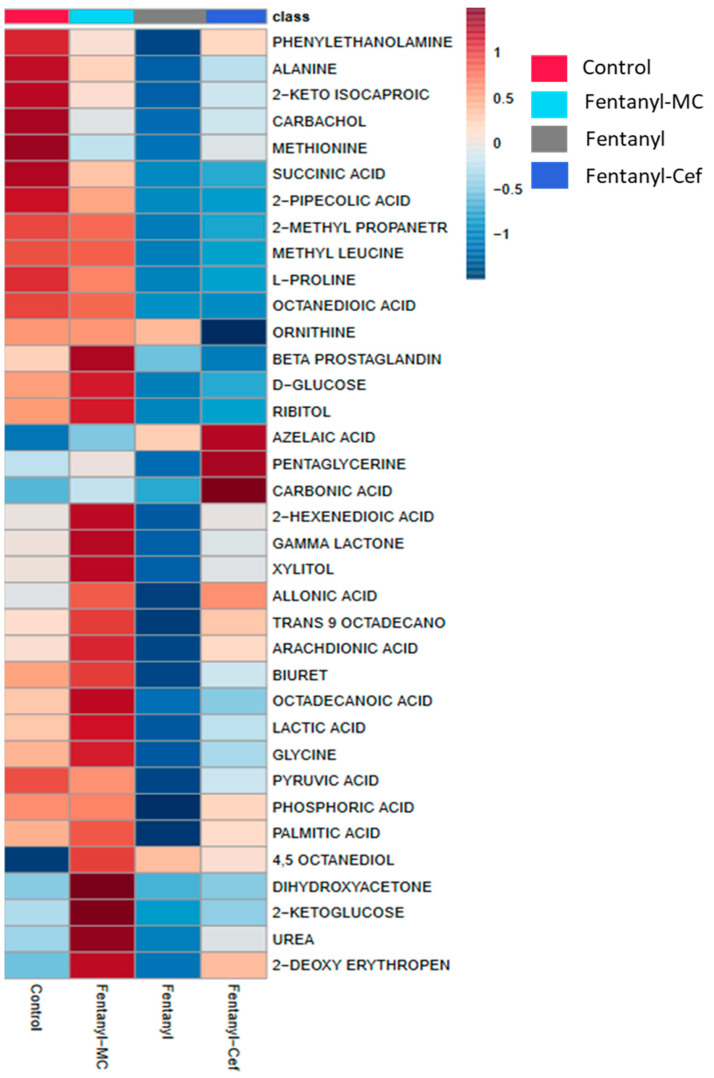
Heatmap analysis model of control, fentanyl, fentanyl–ceftriaxone and fentanyl-MC-100093 metabolomic profiles. Cef, ceftriaxone; MC, MC-100093.

The metabolite heatmap represents the changes in metabolites with each class (drug). Red denotes a rising trend of metabolites, whereas blue denotes a decreasing trend of metabolites. Heatmap analysis showed overall differences in the metabolomic profiling of several metabolites between the groups. MC-100093 treatment showed superior results in modulating many metabolites, such as azelaic acid, *l*-proline, methyl leucine, phosphoric acid, pyruvic acid, and other metabolites in mice treated with fentanyl overdose as compared to ceftriaxone treatment (Figure 3). However, ceftriaxone was also able to modulate metabolites in fentanyl-overdosed mice, including gamma lactone, xylitol, and palmitic acid (Figure 3).

#### 3.1.3. Partial Least-Squares-Discriminant Analysis (PLSDA) of Fentanyl, Fentanyl–Ceftriaxone, and Fentanyl-MC-100093 Groups

**Figure 4:**
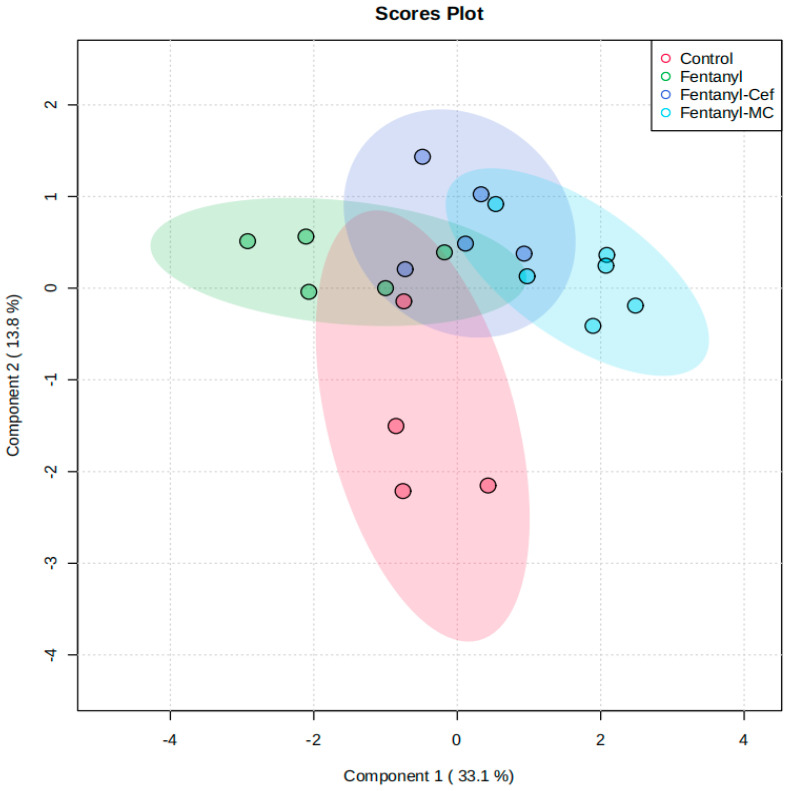
Partial least-squares-discriminant analysis (PLS-DA) model control, fentanyl, fentanyl–ceftriaxone and fentanyl-MC-100093 metabolomic profiles. *n* = 4–6/group. Cef, ceftriaxone; MC, MC-100093.

We further performed PLS-DA modeling of the overall metabolites among the four groups. The analysis revealed some segregation between the groups (Figure 4).

#### 3.1.4. Peak Ratio of Selected Metabolites of Fentanyl, Fentanyl–Ceftriaxone and Fentanyl-MC-100093 Groups

**Figure 5:**
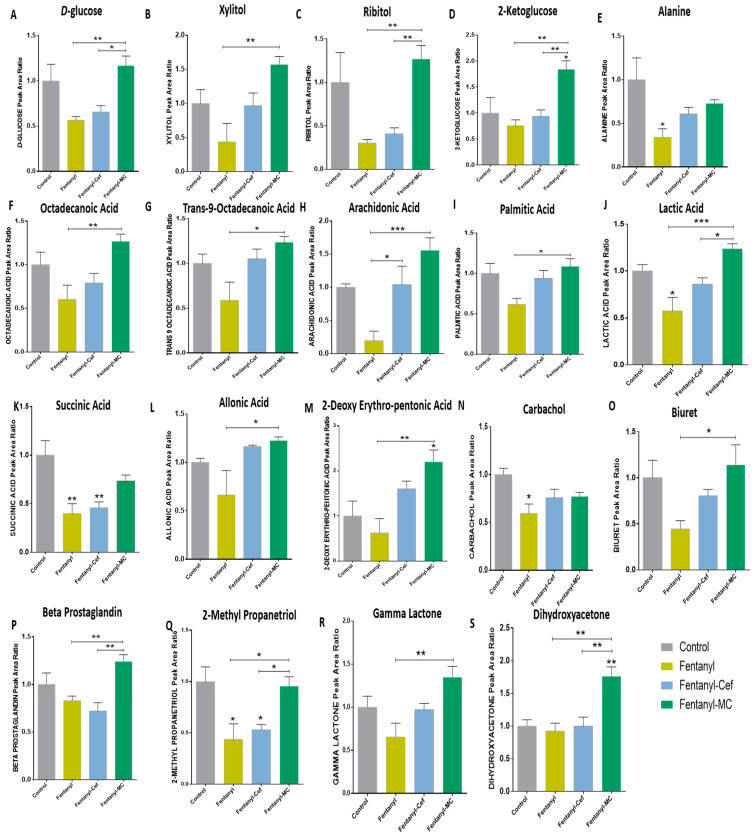
Effects of ceftriaxone and MC-100093 on selected metabolites in liver of fentanyl-overdosed mouse model. (**A**) One-way ANOVA followed by Holm–Sidak’s multiple comparisons test showed that the MC-100093-fentanyl group showed higher *d*-glucose compared to fentanyl and the fentanyl-cef groups. (**B**) One-way ANOVA followed by Holm–Sidak’s multiple comparisons test showed that fentanyl-MC-100093 had higher xylitol compared to the fentanyl group. (**C**) One-way ANOVA followed by Holm–Sidak’s multiple comparisons test showed that MC-100093-fentanyl had higher ribitol compared to the fentanyl and fentanyl-cef groups. (**D**) One-way ANOVA followed by Holm–Sidak’s multiple comparisons test showed that fentanyl- MC-100093 had higher xylitol compared to thew fentanyl group. (**E**) One-way ANOVA followed by Holm–Sidak’s multiple comparisons test showed lower alanine in the fentanyl group compared to controls. (**F**) One-way ANOVA followed by Holm–Sidak’s multiple comparisons test showed that octadecanoic acid was higher in fentanyl-MC-100093 compared to the fentanyl group. (**G**) One-way ANOVA followed by Holm–Sidak’s multiple comparisons test showed that trans-9 octadecanoic acid was higher in fentanyl-MC-100093 compared to the fentanyl group. (**H**) One-way ANOVA followed by Holm–Sidak’s multiple comparisons test showed that arachidonic acid was lower in the fentanyl group compared to the fentanyl–ceftriaxone and fentanyl-MC-100093 groups. (**I**) One-way ANOVA followed by Holm–Sidak’s multiple comparisons test showed that palmitic acid was lower in the fentanyl group compared to the fentanyl-MC-100093 group. (**J**) One-way ANOVA followed by Holm–Sidak’s multiple comparisons test showed lower lactic acid in the fentanyl group compared to controls; however, lactic acid was higher in the fentanyl-MC-100093 group compared to the fentanyl and fentanyl–ceftriaxone groups. (**K**) One-way ANOVA followed by Holm–Sidak’s multiple comparisons test showed that succinic acid was lower in the fentanyl and fentanyl–ceftriaxone groups compared to the control group. (**L**) One-way ANOVA followed by Holm–Sidak’s multiple comparisons test showed that allonic acid was lower in the fentanyl group compared to the fentanyl-MC-100093 group. (**M**) One-way ANOVA followed by Holm–Sidak’s multiple comparisons test showed that 2-deoxy erythro-pentonic acid was higher in fentanyl-MC-100093 compared to the control and fentanyl groups. (**N**) One-way ANOVA followed by Holm–Sidak’s multiple comparisons test showed that carbachol was lower in the fentanyl group as compared to the control group. (**O**) One-way ANOVA followed by Holm–Sidak’s multiple comparisons test showed that biuret was higher in the fentanyl-MC100093 group compared to the fentanyl group. (**P**) One-way ANOVA followed by Holm–Sidak’s multiple comparisons test showed that beta-prostaglandin was higher in the fentanyl-MC-100093 group compared to the fentanyl and fentanyl–ceftriaxone groups. (**Q**) One-way ANOVA followed by Holm–Sidak’s multiple comparisons test showed that 2-methylpropanetriol was lower in the fentanyl and fentanyl–ceftriaxone groups compared to the control and fentanyl-MC-100093 groups. (**R**) One-way ANOVA followed by Holm–Sidak’s multiple comparisons test showed that gamma lactone was higher in the fentanyl-MC-100093 group compared to the fentanyl group. (**S**) One-way ANOVA followed by Holm–Sidak’s multiple comparisons test showed that dihydroxyacetone was higher in the fentanyl-MC-100093 group compared to all other groups. The symbol of statistical significance is shown on any group's bar when it was compared to the control group. Data are reported as mean ± SEM. (* *p* < 0.05, ** *p* < 0.01, *** *p* < 0.001, *n* = 4–6/group). Cef, ceftriaxone; MC, MC-100093.

We evaluated the effects of fentanyl, fentanyl–ceftriaxone, and fentanyl-MC-100093 on selected metabolites such as sugars that were significantly changed between groups using one-way ANOVA followed by Holm–Sidak multiple comparisons (Figure 5). Using Holm–Sidak’s multiple comparisons test, the analysis showed non-significant lower *d*-glucose (Figure 5A), xylitol (Figure 5B) and ribitol (Figure 5C) in the fentanyl and fentanyl–ceftriaxone(but not with xylitol) groups compared to the control group, and that MC-100093 restored these effects in a group exposed to fentanyl overdose. In addition, we found increases of 2-ketoglucose peak area ratios in the fentanyl-MC-100093 group as compared to the other three groups (Figure 5D). We also determined the concentrations of selected amino acids such as alanine, methionine, glycine, l-proline, and methyl leucine in livers of the fentanyl, fentanyl–ceftriaxone, and fentanyl-MC-100093 groups. One-way ANOVA showed significant differences only in alanine, methyl leucine, and *l*-proline among the groups, and Holm–Sidak multiple analysis showed that alanine was lower in the fentanyl group than in the control group (Figure 5E). There were no significant differences in methionine (Figure S1A), glycine (Figure S1B), *l*-proline (Figure S1C), methyl leucine (Figure S1D), or phenylethanolamine (Figure S1E) among the groups.

We explored the effects of fentanyl, fentanyl–ceftriaxone, and fentanyl-MC-100093 on selected fatty acids such as octadecanoic acid, trans-9 octadecanoic acid, arachidonic acid, and palmitic acid. One-way ANOVA revealed significant differences in the selected fatty acids. Holm–Sidak’s multiple comparisons test showed that octadecanoic acid (Figure 5F) was higher in the fentanyl-MC-100093 group than in the fentanyl group. Trans-9 octadecanoic acid (Figure 5G) peak area ratios were higher in the fentanyl-MC100093 group than in the fentanyl group. Arachidonic acid (Figure 5H) was lower in the fentanyl group than in the ceftriaxone and MC-100093 groups, while palmitic acid (Figure 5I) was lower in the fentanyl group than in the MC-100093 group.

We investigated the effects of fentanyl, fentanyl–ceftriaxone, and fentanyl-MC-100093 on selected acids. One-way ANOVA showed significant differences in lactic acid, succinic acid, and allonic acid peak area ratios among the groups. Holm–Sidak’s multiple comparison test showed that lactic acid (Figure 5J) was lower in the fentanyl group than in the control group; however, lactic acid was higher in the fentanyl-MC-100093 group than in the fentanyl group. Fentanyl-MC-100093 group showed higher lactic acid peak area ratios than the fentanyl–ceftriaxone group did. Statistical analysis showed that succinic acid (Figure 5K) was lower in the fentanyl and fentanyl–ceftriaxone groups than in the control group. The analysis showed that allonic acid peak area ratio was lower in the fentanyl group than in the fentanyl-MC-100093 group (Figure 5L). One-way ANOVA showed no significant differences in phosphoric acid (Figure S1F), 2-pipecolic acid (Figure S1G), and carbonic acid (Figure S1H) among the groups.

We further investigated the effects of fentanyl, fentanyl–ceftriaxone, and fentanyl-MC-100093 on 2-hexenedioic, 2-ketoisocaproic, 2-deoxyerythropentoic, octanedioic, azelaic, and pyruvic acids. One-way ANOVA showed a significant difference in 2-deoxyerythropentoic acid peak area ratios among the groups. Holm–Sidak’s multiple comparisons test showed that 2-deoxy erythro-pentonic acid (Figure 5M) was higher in the fentanyl-MC-100093 group than in the control and fentanyl groups. One-way ANOVA showed no significant differences in 2-hexenedioic acid (Figure S1I), 2-ketoisocaproic acid (Figure S1J), octanedioic acid (Figure S1K), azelaic acid (Figure S1L), or pyruvic acid (Figure S1M) among the groups.

We further determined the effects of fentanyl, fentanyl–ceftriaxone, and fentanyl-MC-100093 on carbachol, pentaglycerine, biuret, urea, and beta-prostaglandins. One-way ANOVA showed significant differences in carbachol, biuret, and beta-prostaglandin peak area ratios among the groups, but not in pentaglycerine (Figure S1N) and urea (Figure S1O) peak area ratios. Holm–Sidak’s multiple comparisons test revealed that carbachol (Figure 5N) was lower in the fentanyl group than in the control group. The analysis also showed that biuret (Figure 5O) was higher in the fentanyl-MC-100093 group than in the fentanyl group. Moreover, the analysis revealed that beta-prostaglandin (Figure 5P) was higher in the fentanyl-MC-100093 group than in fentanyl and fentanyl–ceftriaxone groups.

Lastly, we investigated the effects of fentanyl, fentanyl–ceftriaxone, and fentanyl-MC-100093 on 2-methylpropanetriol, ornithine, gamma-lactone, dihydroxy acetone, and 4,5 octanediol. One-way ANOVA showed significant differences in 2-methylpropanetriol, gamma-lactone, and dihydroxy acetone among the groups, but not in ornithine (Figure S1P) and 4,5 octanediol (Figure S1Q). Holm–Sidak’s multiple comparisons test showed that 2-methylpropanetriol (Figure 5Q) was lower in the fentanyl and fentanyl–ceftriaxone groups than in the control and fentanyl-MC-100093 groups. The analysis also revealed that the gamma lactone peak area ratio (Figure 5R) was higher in the fentanyl-MC100093 group than in the fentanyl group. Moreover, dihydroxyacetone peak area ratio (Figure 5S) was higher in the fentanyl-MC100093 group than in the other groups.

### 3.2. Enrichment Analysis Model of Control, Fentanyl, Fentanyl–Ceftriaxone, and Fentanyl-MC-100093 Metabolomic Profiles

**Figure 6:**
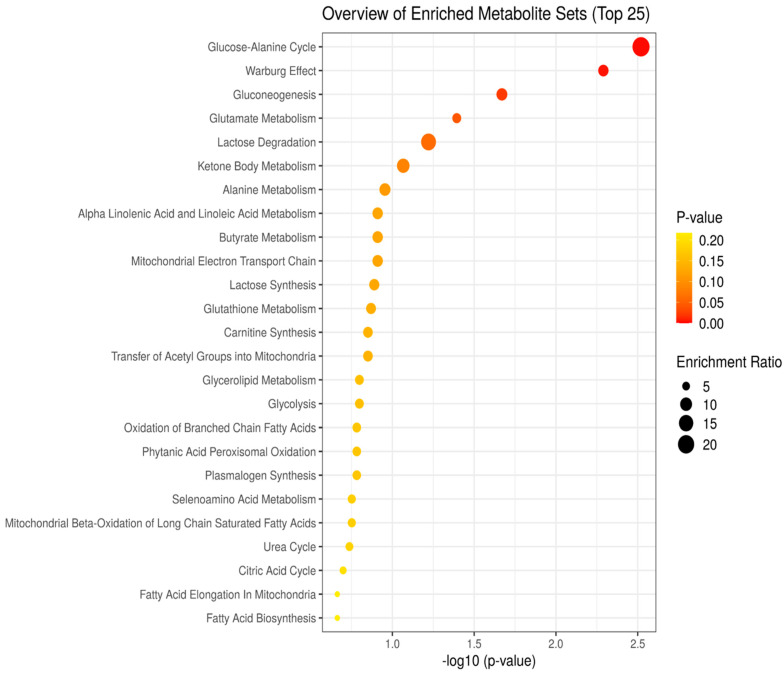
Overview of top 25 enriched metabolite pathways ordered based on *p* value and enrichment ratio.

The top 25 enriched metabolite pathways were based on the p value and enrichment ratios were generated using the MetaboAnalyst software. The analysis showed that the most significant pathways involved in the altered metabolites were the glucose–alanine cycle, Warburg effect, gluconeogenesis, glutamate metabolism, lactose degradation, and ketone body metabolism (Figure 6).

### 3.3. Histopathology Analysis and Protein Expression of IL-6 and CYP-3A4 in Mouse Model of Fentanyl Overdose Treated with Beta-Lactams

**Figure 7:**
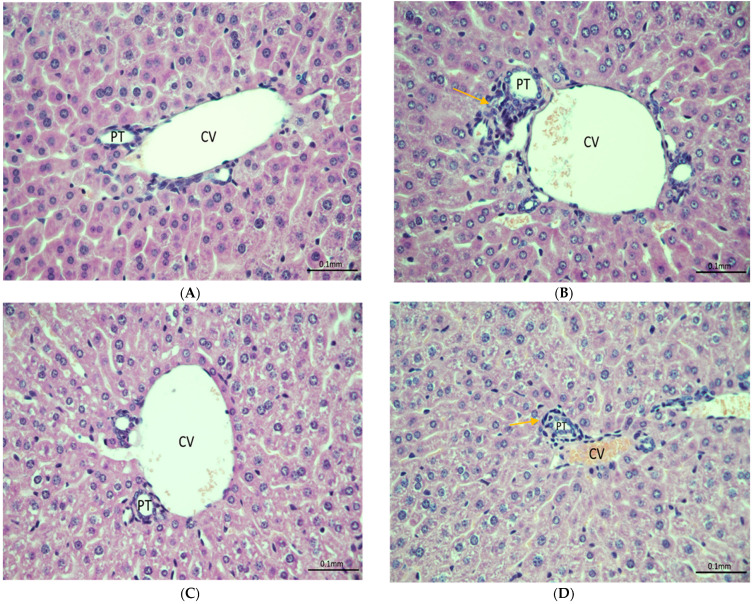
(**A**) Section of liver showing normal portal tract and central vein with surrounding unremarkable hepatocytes in the control group. (**B**) Section of liver obtained from the fentanyl-overdose-treated group showing inflammation in portal tract and central vein. (**C**) Section of liver showing limited portal tract inflammation in the fentanyl–ceftriaxone group indicating the protective effects of ceftriaxone against fentanyl overdose. (**D**) Liver tissue showing minimal to mild inflammation around a bile duct in the fentanyl-MC100093 group. Yellow arrows indicate inflammation. H/E stain ×400. Cef, ceftriaxone; MC, MC-100093; PT, Portal tract; CV, Central vein.

Liver injury was investigated using histopathological analysis. Moderate liver injury was observed in liver sections of mice exposed to fentanyl overdose (Figure 7B) as compared to the control group (Figure 7A); a protective effect was introduced with ceftriaxone pre-treatments (Figure 7C). Minimal to mild inflammation was found in liver sections of the fentanyl-MC100093 group (Figure 7D).

### 3.4. Liver Protein Expression of IL-6 and CYP-3A4 in Mouse Model of Fentanyl Overdose Treated with Beta-Lactams

**Figure 8:**
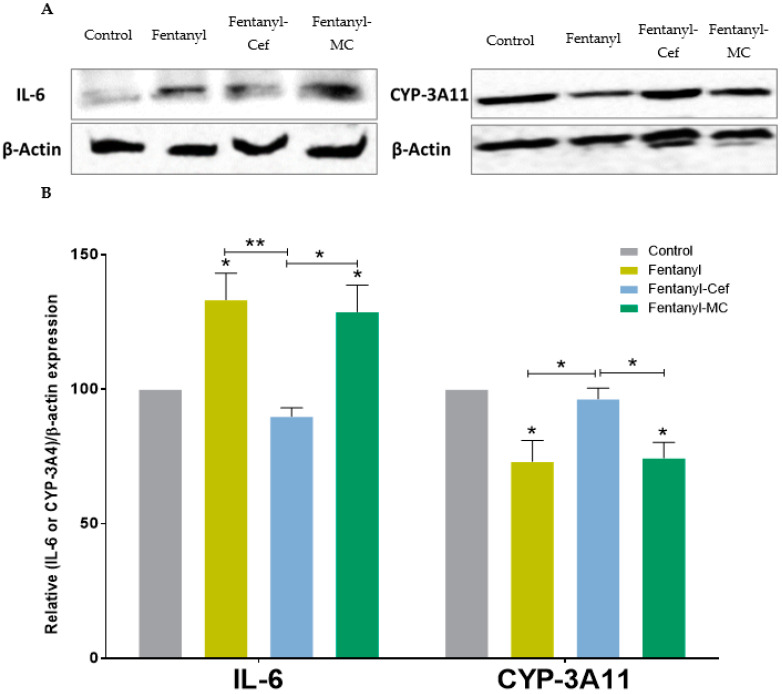
(**A**) IL-6 and CYP3A11 (mouse homolog of human CYP3A4) bands expression in the control, fentanyl, fentanyl–ceftriaxone, and fentanyl-MC100093 groups. (**B**) One-way ANOVA followed by Holm–Sidak’s multiple comparisons test showed that liver IL-6 expression was increased in the fentanyl and fentanyl-MC100093 groups compared to the control and fentanyl–ceftriaxone groups; moreover, liver CYP3A11 expression was lower in the fentanyl and fentanyl-MC-100093 groups compared to the control and fentanyl–ceftriaxone groups (*n* = 4/group). Data are reported as mean ± SEM. (* *p* < 0.05, ** *p* < 0.01). Cef, ceftriaxone; MC, MC-100093.

Protein expression study showed that fentanyl overdose increased IL-6 expression and decreased CYP-3A11 expression in the liver as compared to the control group. These effects on IL-6 and CYP-3A4 were normalized with ceftriaxone, but not with MC100093 (Figure 8A,B).

## 4. Discussion

In the current study, we evaluated the metabolomic profiles in the liver of a fentanyl overdose mouse model, and investigated whether treatment with beta-lactams (ceftriaxone and MC-100093) could modulate any changes in these metabolomic profiles. Using GC-MS, we found that fentanyl overdose reduced several metabolites. Moreover, MC-100093 and ceftriaxone restored some metabolites in fentanyl-overdosed mice, and MC-100093, which does not possess antimicrobial properties, exhibited superior effects. Additionally, enrichment pathway analysis showed that the glucose–alanine cycle, Warburg effect, gluconeogenesis, glutamate metabolism, lactose degradation, and ketone metabolomics pathways were involved in metabolites that were affected by fentanyl overdose and normalized by beta-lactam treatments.

The effects of opioids extend well beyond the mere reduction of pain and may even influence how the body processes sugar. The effect of opioid overdose, particularly tramadol overdose, on blood glucose concentration was reported in a previous study [39]. Although a low dose of tramadol for 13 days did not affect blood glucose concentrations, a tramadol overdose slightly reduced blood glucose concentrations [39]. However, opioid exposure has been associated with increased blood glucose and decreased insulin concentrations [40]. In addition, patients addicted to opioids tend to have lower glucose tolerance, which suggests that there might be an association between opioids and glucose metabolism [40]. Our study investigated the effects of fentanyl overdose on liver sugars, indicating that the amount of drug taken and the target organ play a crucial role in the concentrations of sugars in fentanyl-overdosed mice. We found that fentanyl overdose induced non-significant reductions in glucose and ribitol peak area ratios in mice compared to the control group, and MC-100093 treatment reversed this effect. The heatmap visualization analysis, with respect to the control group, showed that MC-100093 reversed the effects of fentanyl overdose on *d*-glucose and ribitol. It is important to note that a recent study showed that chronic ethanol exposure increased GLT-1 expression in the liver, and that ceftriaxone and MC-100093 attenuated this effect [32,33]. An increase in liver GLT-1 expression might be associated with elevated intracellular glutamate concentrations in the liver, an effect associated with increased insulin secretion [41]. Additionally, pretreatment with MC-100093 in mice exposed to fentanyl overdose increased the peak area ratios of other sugars (e.g., ketoglucose and xylitol) as compared to the fentanyl overdose group. These findings provide information on the modulatory effects of MC-100093 on liver sugar peak area ratios.

Accordingly, our enrichment analysis showed that the glucose–alanine cycle, Warburg effect, gluconeogenesis, and lactose degradation, as part of the glucose metabolism pathways, were the most significantly affected by fentanyl overdose when compared to other metabolic pathways. Enrichment data also showed that glutamate metabolism was highly affected by fentanyl overdose. Further studies are warranted to investigate the role of each factor, including the type of drug, dose, and site of action, on the sugars.

In the analysis of the selected amino acids, only alanine was found to be significantly decreased in the fentanyl overdose group compared to the control group. Alanine plays an important role in the breakdown of muscle proteins in order to obtain more glucose for the generation of additional adenosine triphosphate (ATP) necessary for muscular contraction (the glucose–alanine cycle), and as an important regulator of glucose metabolism, a deficiency in alanine may cause impairment in these pathways [40,42]. Alanine is converted into glucose in the liver, and it was found to be decreased in the liver of fentanyl overdose mice. Our study also found that beta-lactam compound fentanyl groups did not show any significant changes in alanine in the liver compared to the control group. We suggest that opioids, at least in terms of fentanyl overdose, affect the glucose–alanine cycle pathway in the liver. In addition to its role in protein synthesis, glutamate is also an amino acid that plays a significant metabolic role in many organisms. Glutamate plays a role in nitrogen assimilation, nucleoside synthesis, cofactor biosynthesis, and secondary natural product formation. Glutamate is also involved in amine catabolism [43]. Therefore, the glutamate metabolic pathways are affected and can be triggered by fentanyl overdose, and GLT-1 upregulators (e.g., MC-100093 and ceftriaxone) can attenuate this effect.

Among the selected fatty acids, all metabolites were significantly different between the control, fentanyl and treatment groups. Our statistical analysis showed that both arachidonic acid and palmitic acid were non-significantly decreased in the fentanyl overdose group and that beta-lactams normalized these effects. Moreover, heatmap visualization analysis, with respect to the control group, revealed that beta-lactams reversed the effects of fentanyl overdose on arachidonic acid and palmitic acid. Arachidonic acid is an essential component of biological cell membranes, providing the flexibility and fluidity required for all cell functions, particularly in the nervous system, immune system, and skeletal muscles [44]. Palmitic acid, which accounts for 20–30% of the total fatty acids in the human body, plays a major role in the formation of cell membranes, signaling molecules, and pulmonary lipid secretions [45]. Therefore, opioid overdose may have negative consequences on the physiological functions of these fatty acids in the body. Our analysis also revealed that the MC-100093-fentanyl group showed higher peak areas of other fatty acids, including octadecanoic acid and trans-9 octadecanoic acid, than the fentanyl overdose group. Octadecanoic acid, also known as stearic acid, is a saturated long-chain fatty acid with an 18-carbon backbone. Trans-9 octadecanoic acid, also called elaidic acid, is an unsaturated trans fatty acid that increases the activity of plasma cholesteryl ester transfer protein, which lowers high-density lipoprotein [46]. Importantly, our enrichment pathway analysis indicates that ketone body metabolism is a pathway that is significantly affected by fentanyl overdose, as shown by changes in certain liver metabolites. When carbohydrates are decreased in supply, ketone bodies are metabolized via evolutionarily conserved pathways that support bioenergetic homeostasis in many organs of the body, such as the heart, brain, and skeletal muscles [47].

**Figure 9:**
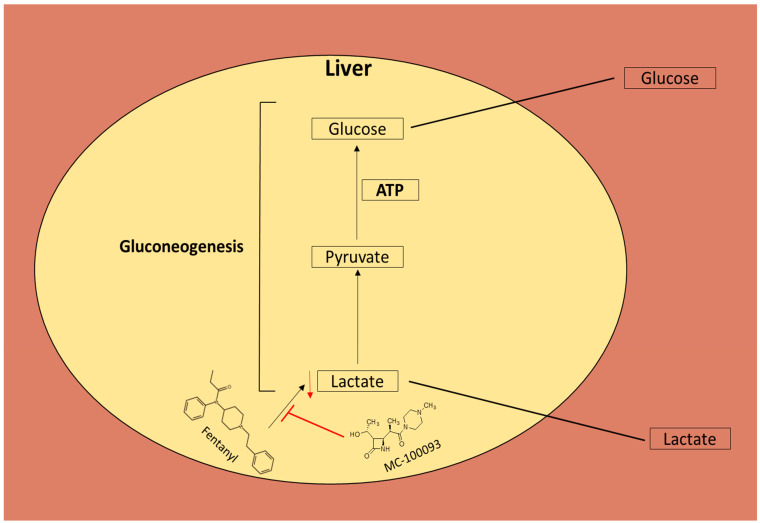
A schematic diagram shows the effects of fentanyl and MC-100093 on lactic acid in the gluconeogenesis pathway. ATP, adenosine triphosphate.

Among other acids, lactic acid and succinic acid were all significantly lower in the fentanyl group than in the control group, and lactic acid was normalized by MC-100093 treatment. Allonic acid was higher in the fentanyl-MC-100093 group than in the fentanyl group. Lactic acid (or lactate) is synthesized from pyruvate by lactate dehydrogenase in the muscle cells, which generates nicotinamide adenine dinucleotide in the same reaction, and then utilized in glycolysis to produce ATP. In hepatocytes, lactic acid is converted to pyruvate for glucose generation with the involvement of ATP, a pathway known as gluconeogenesis [48]; however, MC-100093 was found to attenuate fentanyl-induced reduction in liver lactic acid (Figure 9). In addition to its important role in ATP generation, studies have shown its roles in other pathological and physiological mechanisms, including ischemic injury, wound healing, immune system tolerance, neuroprotection, memory formation, and cancer growth and metastasis [49,50]. Succinic acid is an important intermediate in the Krebs cycle and serves as an electron donor in the production of fumaric acid and the reduced form of flavin adenine dinucleotide; a lower concentration may indicate an impairment in this process [51,52]. Dihydroxyacetone (DHA) is primarily used as an ingredient in sunless tanning. It is often derived from plant sources, such as sugar beets and sugar cane, and by the fermentation of glycerin. DHA can be used in cosmetic manufacturing, such as masking hypopigmented vitiligo macules, and may provide UVA protection [53]. In our study, the fentanyl-MC-100093 group showed higher peak areas of DHA in the liver than the other groups. Moreover, MC-100093 attenuated the effect of fentanyl-induced decrease in 2-methyl propanetriol in the liver. We also found that carbachol peak area ratios were lower in the fentanyl group as compared to the control group. There was also a non-significant trend of normalizing the peak area ratios of *l*-proline, palmitic acid, methyl leucine, and pyruvic acid with MC-100093 treatment in the fentanyl overdose mouse model.

Repeated exposure to fentanyl increased serum glutamic–pyruvic transaminase (SGPT) in rats [54], and this increase may lead to liver damage. Moreover, ischemic-like damage in the liver was observed in rats exposed to 4-fluoro-isobutyrylfentanyl, an analogue of fentanyl [55]. Fentanyl was also found to increase both SGPT and serum glutamic oxaloacetic transaminase in rats with and without liver cirrhosis [56]. Using histopathology approach, we found that fentanyl overdose increased inflammation in mouse liver, and this effect was protected against with ceftriaxone pre-treatment. Minimal to mild inflammation was observed in the fentanyl overdose group pre-treated with MC-100093. These findings were consistent with our protein expression study where we found that IL-6 was increased in the liver of the fentanyl overdose group as compared to the control and fentanyl–ceftriaxone groups. In addition, fentanyl overdose decreased liver CYP 3A4 expression, an effect restored with ceftriaxone, but not MC-100093. It is important to note that inflammation is associated with reduced gene and protein expression of CYP3A4 in hepatocytes [57,58]. The protective effect observed mainly from ceftriaxone is due to the fact that the dose used for this drug is optimal; however, the dose used for MC-100093 was on the lower side. Further studies are warranted to test the higher doses of MC-100093 against the effects of fentanyl overdose in the liver.

In conclusion, the heatmap analysis of the metabolomics represents a visual summary of the metabolites affected by fentanyl overdose, and whether ceftriaxone or MC-100093 normalized it. MC-100093 reversed many metabolites that were affected by fentanyl overdose, which shows a promising potential for the treatment of metabolic abnormalities induced by opioid overdoses. The changes in liver metabolites induced by fentanyl overdose were associated with liver inflammation, an effect attenuated with beta-lactam pre-treatments. However, further molecular metabolomic studies are needed to investigate the effects of fentanyl overdose and beta-lactams on metabolic pathways at the gene and protein states. Future studies are also warranted to investigate the effects of repeated exposure to, and the overdose, reinstatement, and withdrawal of, fentanyl on metabolites using an untargeted metabolomic approach to determine the most sensitive metabolites. We suggest here that based on our previous study on drugs of abuse, ceftriaxone and MC-100093 are considered protective against drugs of abuse-induced liver injury [32]). Studies are warranted to validate our findings and explore the therapeutic effects of ceftriaxone and MC-100093 after fentanyl overdose in the liver. Further studies are also warranted to investigate the effects of ceftriaxone and MC-100093 alone in overall liver metabolites to confirm the protective effect of these beta-lactams. Investigating the systemic effects of fentanyl overdose and beta-lactam treatments on metabolites and inflammatory markers warrant investigation in future studies.

## Data Availability

The data presented in this study are available within the article.

## Supplementary Materials

The following supporting information can be downloaded at: https://www.mdpi.com/article/10.3390/metabo13080965/s1. Figure S1: One-way ANOVA test followed by Holm-Sidak’s multiple comparisons showed non-significant differences in other metabolites between the groups.
