# Supplementary material for: Liver Metabolomics and Inflammatory Profiles in Mouse Model of Fentanyl Overdose Treated with Beta-Lactams
Source: Metabolites. 2023 Aug 21;13(8):965. doi: 10.3390/metabo13080965 (PMC10456707; doi:10.3390/metabo13080965)
Supplement: Supplementary file 1 [file metabolites-13-00965-s001.zip › metabolites-2515106-supplementary.pdf]

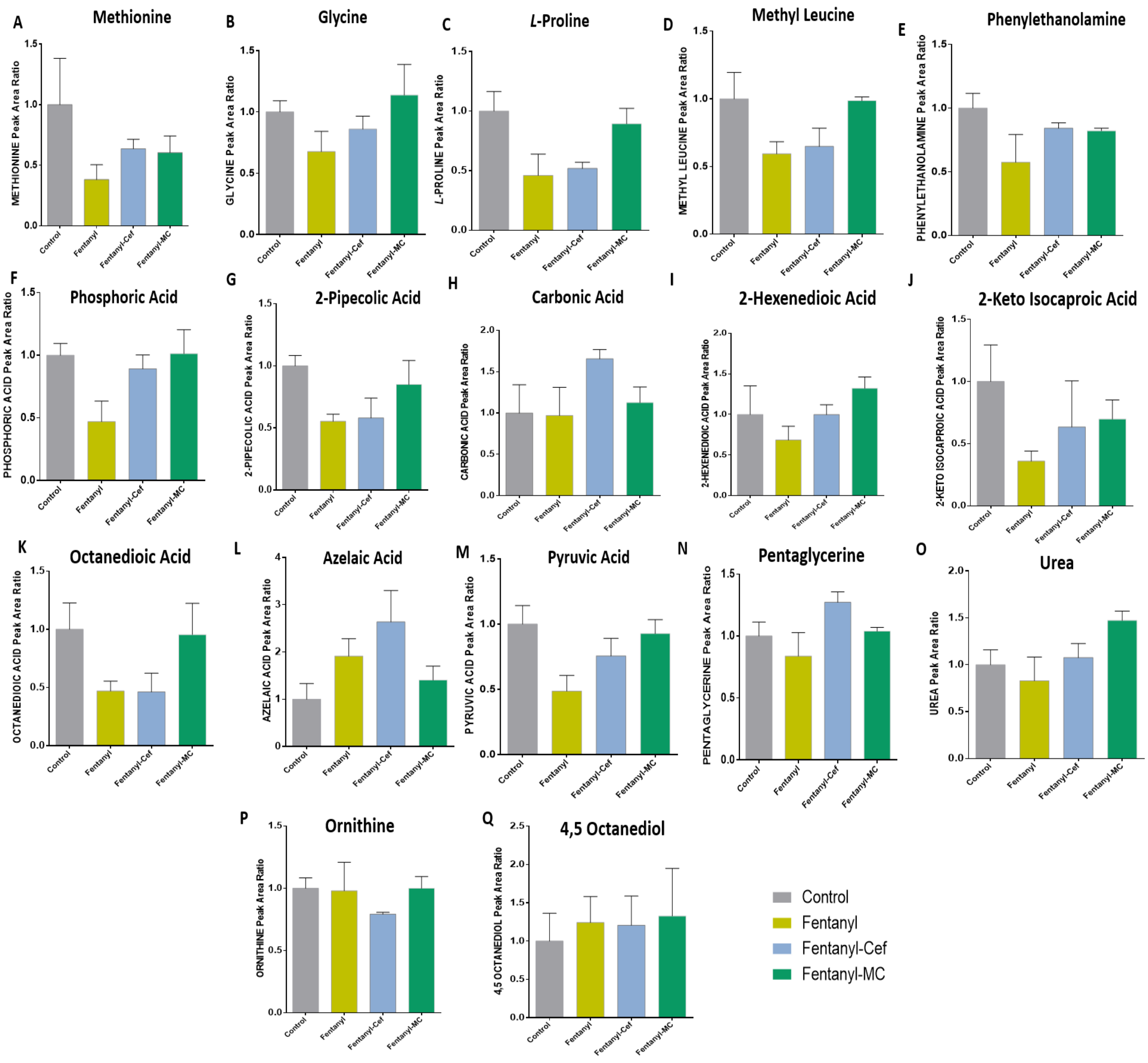

Figure S1. One-way ANOVA test followed by Holm-Sidak's multiple comparisons showed non-significant differences methionine (A), glycine (B), *l*-proline (C), methyl leucine (D), phenylethanolamine (E), phosphoric acid (F), 2-pipecolic acid (G), carbonic acid (H), 2-hexenedioic acid (I), 2-keto isocaproic acid (J), octanedioic acid (K), azelaic acid (L) , pyruvic acid (M), pentaglycerine (N), urea (O), ornithine (P) and 4,5 octanediol (Q) between the groups. Data are reported as mean ± SEM. Cef, ceftriaxone; MC, MC-100093.
